# Real-Time Wearable sEMG Onset Detection and Phase Discrimination of Sit-to-Stand Movement via a Compact Dual-Channel DD-CNN

**DOI:** 10.3390/s26144375

**Published:** 2026-07-10

**Authors:** Meernah Mohammed Alabdullah, Aiqin Liu, Yiliu Tu, Sheng Quan Xie

**Affiliations:** 1 Biomedical Engineering Department, Imam Abdulrahman Bin Faisal University, Dammam 31451, Saudi Arabia; mmmalabdullah@iau.edu.sa; 2 School of Electronic and Electrical Engineering, University of Leeds, Leeds LS2 9JT, UK; 3 School of Biomedical Sciences, University of Leeds, Leeds LS2 9JT, UK; 4 Modern College of Engineering, Henan Normal University, Xinxiang 453007, China; tuyiliu@htu.edu.cn

**Keywords:** surface electromyography, onset/offset detection, sit-to-stand, stand-to-sit, movement phase classification, convolutional network, wearable sensing system, real-time processing, embedded deep learning

## Abstract

Repeated sit-to-stand and stand-to-sit transitions load the knee extensors and may contribute to work-related musculoskeletal disorders. Reducing this load requires assistive devices and monitoring of knee function, which depend on real-time onset/offset detection and direction-aware classification of each transition. However, no prior wearable surface electromyographic system has delivered this capability for real-time. This study presents a deep learning method that computes both onset/offset detection and direction discrimination of sit-to-stand and stand-to-sit in a developed wearable surface electromyographic system in real-time. Two ESP32-S3 nodes and a hub record from the vastus lateralis and vastus medialis and run a per-burst convolutional detector, while the hub runs a dual-branch classifier with seventeen handcrafted features. Trained offline on the public Gait120 dataset, the networks are deployed unchanged with embedded-firmware parity to the MATLAB reference. Under leave-one-subject-out evaluation on Gait120, the offline classifier separated each transition with 99.6% accuracy and the detector achieved 96.6% completeness. In real-time recordings from thirty healthy adults, the system retained 85.6% classification and 82.0% detection accuracy, with ≈100 ms latency and a 618 KB network footprint. Results show that a low-power wearable delivers combined detection and phase discrimination in real-time, supporting the potential application in assistive-device control and knee-joint monitoring.

## 1. Introduction

Repeated sit-to-stand (STS) and stand-to-sit (StS) movements are among the most mechanically demanding postural movements of daily and occupational life. These transitions are performed many times each shift in tasks such as assembly work, machine operation, nursing and patient handling, and the place repeated load on the knee extensors. Within the quadriceps femoris, the vastus lateralis (VL) and the vastus medialis (VM) are the two superficial primary agonists of this group, where they generate the concentric extension that lifts the body during STS and remain concentric extension that lifts the body during STS and remain active eccentrically during the lowering phase of StS to control the descent [1,2]. Both muscles lie along the anterior thigh and are accessible from electrode sites standardized by Surface Electromyography for the Non-Invasive Assessment of Muscles (SENIAM), which makes them tractable targets for two-channels electromyographic (sEMG) recording without specialist instrumentation [3]. Their relative activation also carries clinical weight, where an altered VL-to-VM ratio is one of the established neuromuscular features of knee osteoarthritis (KOA), where structural and electromyographic studies reported that the imbalance correlates with the presence and degree of knee joint abnormalities and with reduced functional capacity in everyday tasks such as rising from chair [4]. Cumulative exposure to such loaded transitions is implicated in the high global burden of work-related musculoskeletal disorders (WMSDs) of the lower limb, which account for a substantial share of years for people lived with disability worldwide [5]. The same loading carries added consequence with age, when reduced quadriceps strength is interdependently associated with an elevated risk of falls in older adults, lengthening transition duration and raising the likelihood of failed attempts [6].

To mitigate this exposure and assist those who cannot complete the transition unaided, lower limb wearable assistive devices such as exoskeletons and powered orthoses that act at the hip and knee have been developed to support the knee extensors during the chair rise, both for occupational use and for clinical rehabilitation [7,8]. Their effectiveness rests on the sensing front-end that drives the controller [9,10]. For a device to provide assistance in a way the wearer accepts, it must be capable of identifying not only the onset and termination of a transition but also distinguishing whether the movement corresponds to an STS or StS activity, because the two impose opposing concentric and eccentric demands on the same muscles and require different assistive responses [1]. Real-time, direction-aware information about each transition is therefore a prerequisite of effective human–machine interaction in such devices, and is equally informative for occupational ergonomic assessment, where the number of transitions performed, timing and direction are exposure variables of direct interest [9,10].

To date, the studies that focused on the detecting and identifying STS and StS transitions have been dominated by inertial measurement units (IMUs) that placed on the trunk, thigh, or pelvis [11,12] and incorporated into wearable systems for occupational biomechanical risk assessment [10]. By exploiting the unambiguous sign of trunk and thigh rotation, IMU-based classifier have separated STS from StS with near-perfect offline accuracy between 89% to 100 % [11]. Yet, using this modality for movement classification have the following limitations, specially for direction-aware assistive control. First, IMUs observe the kinematic outcomes of the transition rather than the neuromuscular drive that produces it, therefore the earliest available trigger is the start of the motion itself, not the muscle activation that precedes it. Consequentially, this is a non-vital loss of lead time for any controller that must intervene during the transition; because the STS is brief, any detection delay directly reduces the control window, the time between detection motion intent and the movement itself, within which a wearable controller can apply assistive torque of simulation [13,14]. Second, an inertial signal becomes ambiguous whenever the user partially stands, sways forward, or pauses mid-rise, because the same kinematic profile can correspond to distinct muscular intents [15,16].

Surface electromyography (sEMG) instead reflects the neuromuscular drive that precedes movement [13,17], and this neural drive can be used to estimate the muscle forces generated during human movement [18]. Shifting the open question from sensing to how that signal is exploited algorithmically in real-time. Further it reflects the underlying motor intent rather than its mechanical outcome. The prior work that used sEMG for STS discrimination application falls into three methodological tracks. The first track focused on using classical signal processing-based onset detector such as: fixed single or double-threshold rules, adaptive thresholds, the Teager–Kaiser energy operator (TKEO), and the generalized likelihood ratio (GLR), which operates on the rectified or whitened envelop and declare an onset when a static crosses a threshold derived from a rest baseline [19,20,21,22]. Collectively, these methods are sensitive to the signal-to-noise ratio (SNR) of the recording, demand hand-tuned thresholds and rest window statics that do not transfer cleanly across subjects or electrode positions, and incur a detection lag that scales with the persistence required to suppress false crossings. They are typically applied offline on workstation hardware after a full signal has been acquired, and consequently expose onset/offset to a controller while the movements is still unfolding [13].

The second track includes learned detectors, which address several of these limitations. The neural muscle activation detector (NMAD) of Khowailed et al. [23] used a recurrent neural network trained on 100 simulated signals and validated on VL and lateras hamstring sEMG from a single runner, reporting detection that outperforms classical TKEO and GLR baseline with absolute latency error of ⩽3 ms, where it improved robustness to varying SNR. On other hand, the Detection of Muscular Activity using Neural Networks (DEMANN) classifier [24] used a single-layer fully connected network trained on simulated signals, and they reported onset/offset mean absolute error (MAE) less than 20 ms on the simulated test set, while the error raised up to 38 ms on real recordings. Both studies, however, were evaluated offline, and they have not been demonstrated continuous on-body operation on an embedded processor.

The third track has applied sEMG more directly to STS as classification target rather than as an onset signal. The iTransformer-DTL framework of Wang et al. [25] decoded STS phases and joint trajectories from a fused sEMG-IMU stream and reported phases classification accuracy above 99% on both healthy adults and stroke patients. The performance is high, yet the demonstration depends on sixteen sEMG channels paired with a seven IMUs, and it was evaluated offline on workstation hardware. Broader sEMG-based lower limb activity recognition follows the same pattern, with high classification accuracy obtained from multi-channel arrays and offline processing rather than from minimal-channel, on-device execution. Conventional sEMG classification typically relies on handcrafted time- and frequency-domain features combined with classifiers such as linear discriminant analysis, support vector machines, k-nearest neighbors, and random forests [26,27,28]. Although these methods are computationally efficient, their performance depends heavily on carefully selected features, and they often generalize poorly across participants and when applied to noisier signals collected using wearable sensors. More recent approaches instead learn temporal patterns directly from the sEMG signal. Temporal convolutional networks (TCNs), for example, use stacked dilated causal convolutions to capture increasingly long temporal dependencies [29]. A compact and quantised TCN, TEMPONet, achieved 49.6% accuracy on the Ninapro hand gesture benchmark while requiring only 70.9 kB of memory and less than 5 ms for inference on an embedded multicore processor [30]. CNN-BiLSTM models combine convolutional feature extraction with a bidirectional LSTM to capture temporal information in both directions across the movement phase. Using 12-channel forearm sEMG, this approach achieved 86.66% accuracy across seventeen hand gestures in Ninapro DB2 [31]. However, both approaches were designed for upper-limb gesture recognition using multi-channel forearm sEMG and focused only on classification. This methodological track confirms that, the sEMG carries enough information to distinguish STS from other movements, although has not yet delivered detection and direction discrimination together on a wearable processor with minimal channel count.

Taken together, no prior sEMG system across the classical, learner, STS and StS classification combined onset/offset detection with STS movement phases discrimination, in real-time, on wearable itself. Motivated by the assistive-device control, rehabilitation, and fall-risk monitoring applications, this study aim to develop and validate, as a proof of concept in healthy adults, a deep learning (DL) method that performs real-time onset/offset detection and STS/StS direction discrimination from a two-channel wearable sEMG system. The validation in clinical populations is left to future work. The developed method includes: (i) a per-burst convolutional detector developed for real-time onset/offset detection from each sEMG channel, with exponential smoothing, hysteresis, persistence and a look-back buffer to improve temporal stability; (ii) a dual-branch convolutional classifier that combines learned signal representations with seventeen amplitude-invariant handcrafted features to discriminate transition direction; and (iii) a parity-verified C implementation that deploys both networks unchanged on an in-house developed two-channel wearable sEMG sensing system. To the author’s knowledge, this is the first method to perform both onset/offset detection and STS/StS direction discrimination from only two knee extensor sEMG channels, on-device in real-time, without auxiliary sensors.

The reminder of this paper is organized as follows: Section 2 which is method and materials, describes the developed in-house sEMG sensing system, the public dataset used for offline training pipeline, the detector and classifier architecture, the firmware export and parity verification, and the real-time evaluation protocol in healthy participants. Section 3 which represent the results, covers the offline performance on the held-out public dataset and the on-device performance during real-time sessions, including computational footprint and end-to-end latency of the deployed pipeline. Section 4 shows the discussion and interpretation of the obtained results, and places these results against the prior work, further is discusses the future work. Section 5 concludes the overall work.

## 2. Materials and Methods

**Figure 1:**
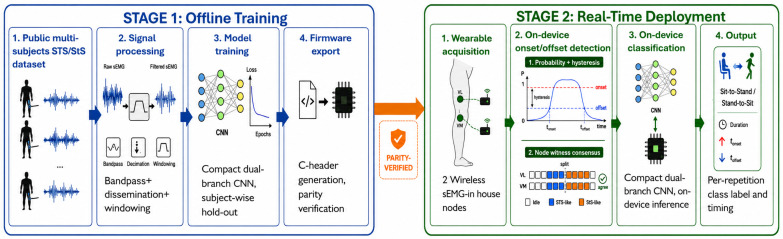
Overview of the developed method, comprising an offline training stage (Stage 1, in blue) and a real-time deployment stage (Stage 2, in green) on the wearable sEMG sensing system, bridged by parity verification of the exported firmware. Arrows show the direction of the processing flow between steps. The abbreviations VL, VM, CNN, STS, and StS denote vastus lateralis, vastus medialis, convolutional neural network, sit-to-stand, and stand-to-sit, respectively; *P* is the per-burst active probability, and $t_{\mathrm{Onset}}$ and $t_{\mathrm{Offset}}$ are the detected onset and offset times. In the node-witness panel, white, blue, and orange boxes denote idle, STS-like, and StS-like bursts. The ellipsis in the dataset panel denotes the remaining subjects of the public multi-subject dataset.

This section provides a detailed description of the method, hereafter referred to as DD-CNN (detection–discrimination CNN), which is divided into two main stages: offline training and real-time deployment, the two stages bridged by parity verification of the firmware export, as shown in Figure 1. For the first stage, it encompasses four steps which are: the public sEMG dataset preparation, signal processing and windowing, dual-branch CNN training with leave-one-subject-out cross-validation, and the export of the network as an embedded C-header. The second stage focuses on real-time execution and comprises four steps: a dual-channel in-house sEMG sensing system acquisition from VL and VM, on-device onset/offset detection, on-device STS/StS phase classification, and real-time output evaluation. Finally, the deployed model is benchmarked against representative existing classification and detection methods under the same evaluation protocol.

### 2.1. Wearable Sensing System Overview

**Figure 2:**
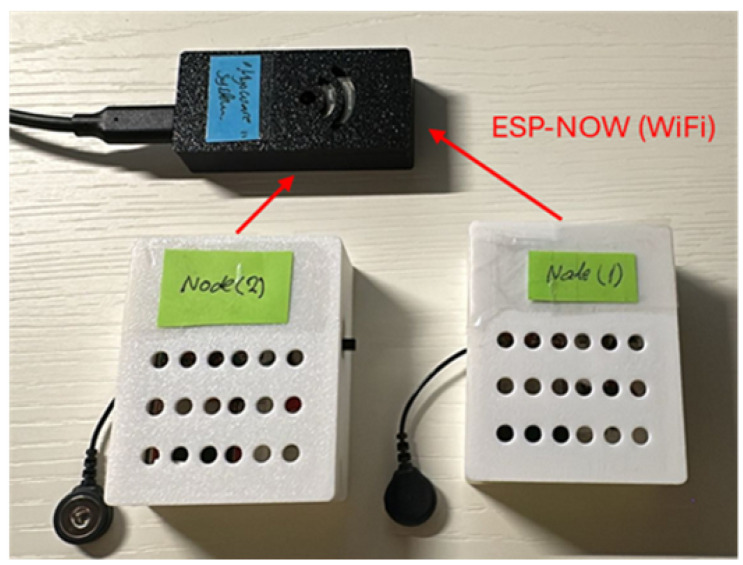
sEMG sensing system prototype.

In the present study, an in-house developed sEMG sensing system prototype was used to detect VM and VL signals during STS and StS activities. The developed sensing system was composed of two wireless sensing nodes, each node encompassing an sEMG MyoWare 2.0 sensor (Advancer Technologies, LLC, Tampa, FL, USA), connected to an ESP32-S3 microcontroller (Espressif Systems, Shanghai, China). The MyoWare 2.0 is a single-differential Ag/AgCl gel electrode sensor, with an on-board instrumentation amplification and first-order 20–500 Hz analogue band-pass filter [32]. This sensor was selected to be used in the present study, because its raw signal has been validated against commercial reference systems such as the Delsys Trigno system [33], and it has been adopted in a wide range of applications, such as muscle fatigue assessment [34], and lower limb activity recognition from the quadriceps [35]. On the other hand, in the present study three microcontrollers were used; two at the sensing node side, and one used as a hub for the acquired data processing, as shown in Figure 2. The microcontroller model, ESP32-S3-WROOM-1, was chosen due to four technical features [36] which are: the dual-core 32-bit Xtensa LX7 processor that is running at 240 MHz; this feature allowed the received sEMG signal to be handled on one core while the onset/offset detection and the CNN inference to be executed on the other core. Moreover, it has an integrated 12-bit successive-approximation analogue-to-digital converter (ADC), used at the sensing node side to digitize the MyoWare raw output without requiring an external ADC. Furthermore, this board offers an integrated 2.4 GHz IEEE 802.11 radio supporting the ESP-NOW peer-to-peer protocol, which allows low-latency wireless communication from the two sensing nodes to the hub without the need for an access point. Even more, the board supports the external high-density flash and pseudo-static RAM that was used on the hub variant to buffer several seconds of dual-channel signal and to store the embedded model weights generated by the exported firmware.

### 2.2. Data Preparation

***Dataset:*** In the present study, the publicly available Gait120 dataset [37] was used to train the model. This dataset encompasses a large number of annotated sit-to-stand (558) and stand-to-sit (569) movements, recorded from healthy adults using laboratory-grade instrumentation and synchronized full-body kinematics. The dataset contains twelve wireless sEMG channels acquired from the right leg; of those, the VL and VM channels were used for training, to match the designed configuration of the current study. Further, each movement is annotated with its movement phase (STS or StS) and temporal boundaries, which served as ground truth for window extraction and labeling. The training labels were derived directly from this dataset: bursts falling within an annotated phase were labeled active, and bursts in the intervening rest intervals were labeled rest.

***Signal Processing:*** Each channel’s data was filtered following the established sEMG signal-processing recommendations [3,38], using a band-pass filter (20–450 Hz) to isolate the dominant sEMG energy band, then a 60 Hz IIR notch filter to remove power line interference. The dataset was recorded at 2 kHz, while the developed wearable sensing system operated at 1 kHz; therefore, the filtered data were down-sampled so that the temporal characteristics of the training data matched those encountered on-device at run-time.

***Window Extraction and Normalization:*** Each phase in the dataset had a variable duration, while the CNN required a fixed-length input. Therefore, each phase was divided into fixed-length analysis windows of 270 samples (270 ms at 1 kHz) using an overlapped sliding-window scheme with 50% overlap, following the same windowing overlap used in [26]. Any phase longer than a single window produced several windows, which both provided fixed-length inputs and augmented the training set. Each channel of each window was then independently normalized by per-window z-scoring. For a window *x* with mean μ and standard deviation σ, the normalized window $\tilde{x}$ was computed using Equation (1).

(1) $$\tilde{x}=\frac{x-\mu }{\sigma }$$

This processing removed the per-subject and per-electrode amplitude scaling that would otherwise dominate the input distribution [28]. It is worth noting that the normalization was applied per window rather than per phase, so that matches the designed firmware, where each window is z-scored immediately before being passed to the network.

***Handcrafted Feature Extraction:*** A set of 17 handcrafted features was computed per phase, then supplied to the model as auxiliary input. These time- and frequency-domain features are well-established in sEMG pattern recognition and remain effective when combined with learned representations [3,26]. In the present study, the features were chosen to be amplitude-invariant, following evidence that amplitude-normalized, spectral, and complexity descriptors are more robust to electrode displacement, gain variation, and inter-session differences than raw-amplitude features [26]. These features span four families: envelope-shape, amplitude-invariant temporal, spectral, and complexity descriptors. These are all summarized in Table 1.

The normalization of the wavelength by the mean absolute value, and using the inter-muscle mean absolute value (MAV) ratio rather than absolute amplitudes, this suppress the amplitude-scaling mismatch between public dataset and the developed wearable recordings, consistent with feature-robustness findings in the sEMG literature [26].

***Partitioning and Class Balancing:*** The dataset was partitioned using leave-one-subject-out cross-validation (LOSO), in which each fold was trained on all subjects except one and tested on the held-out subject, so the reported performance reflects generalization to unseen individuals. To mitigate the residual class imbalance between the two movement types, the training set of each fold was class-balanced by random oversampling of the minority class, complemented by a class-weighted loss during training. Signal- and feature-level normalization statistics were computed exclusively on the training subset of each fold and applied unchanged to the held-out test subject and to the deployed device, preventing information leakage.

### 2.3. DD-CNN: Onset/Offset Detection and Phase Classification

**Figure 3:**
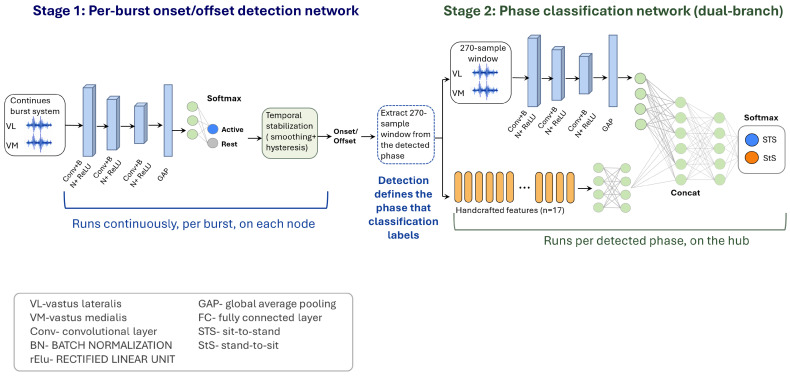
DD-CNN architecture overview, composed of two stages. Stage 1 runs continuously on each node, and Stage 2 runs per detected phase on the hub. Blue rectangles denote convolutional blocks (Conv+BN+ReLU), green circles denote fully-connected units, and orange bars denote the handcrafted feature values. Dashed boxes indicate data passed between the stages, such as the extracted 270-sample window. The coloured output nodes indicate the predicted class (Active or Rest for detection, STS or StS for classification). The ellipsis in the handcrafted-feature row denotes the remaining features up to the full set of seventeen.

The developed system includes a detection network that produces the onset/offset of each phase and a classification network that labels each detected phase as STS or StS. The classification network was designed to be dual-branch, combining a convolutional branch with a fully connected feature branch. This design separated detection from classification, allowing each network to be specialized to its task: the detection network operates per burst in real-time on each wearable sensing node, whereas the classification network operates once per detected phase on the hub. Figure 3 provides an overview of the network architecture design. The developed model and its training were carried out offline in MATLAB R2021a (MathWorks, Natick, MA, USA) on a workstation with an Intel Core i7-1165G7 processor (2.80 GHz) and 16 GB memory; no dedicated GPU was used, due to the compact size of the networks. At the training step, the Adam optimizer with a cross-entropy loss was used to train both networks, and the training was stopped early according to the validation performance to limit overfitting. The normalization statistics were taken from the training set alone, and the resulting weights were exported unchanged for on-device inference.

***Stage 1: Per-burst onset/offset detection:*** Onset and offset are detected with a learned per-burst classifier rather than with a fixed signal processing rule. Each wearable sensing node runs a compact CNN on every incoming burst of the two-channel stream. The network consists of a small stack of 1D convolutional blocks, as shown in Figure 3, each comprising a convolution, batch normalization, and a rectified linear unit. This architecture is followed by global average pooling (GAP) and a softmax layer that outputs the probability of the burst being active rather than rest. At the movement boundaries, the per-burst classifier is inherently noisy, as individual bursts can be misclassified and cause the detected state to flicker. Therefore, the raw per-burst output was stabilized before being used to declare boundaries. The active probability was smoothed across bursts with an exponential moving average using Equation (2).

(2) $$p_{k}=\alpha {\hat{p}}_{k}+\left(1-\alpha \right)p_{k-1}$$

where; ${\hat{p}}_{k}$ is the network’s active probability for burst *k* and α∈ (0,1) that controls the degree of smoothing. The smoothed probability was then converted into onset/offset events through a two-threshold (hysteresis) rule with persistence [19], whereby an onset was declared only after the probability had exceeded an upper threshold $\theta_{\mathrm{On}}$ for $N_{\mathrm{On}}$ of consecutive bursts, and an offset only after it had fallen below a lower threshold $\theta_{\mathrm{Off}}$ for $N_{\mathrm{Off}}$ of consecutive bursts, using Equation (3).

(3) $$\begin{array}{cc} \mathrm{onset}\;\mathrm{at}\;k & ⟺p_{j}>\theta_{\mathrm{on}}\;\left(j=k-N_{\mathrm{on}}+1,\ldots ,k\right),\\ \mathrm{offset}\;\mathrm{at}\;k & ⟺p_{j}<\theta_{\mathrm{off}}\;\left(j=k-N_{\mathrm{off}}+1,\ldots ,k\right) \end{array}$$

where $\theta_{\mathrm{Off}}<\theta_{\mathrm{On}}$. However, the implementation of the dual-threshold hysteresis band mitigates state oscillation when the probability values fluctuate marginally. At the same time, a persistence condition is applied to filter out transient, spurious signal activity. Because this persistence requirement inherently introduces a systematic delay in onset detection, a look-back buffer of recent signal bursts is maintained. By prepending this buffered data to the identified active phase, the system accurately captures the true temporal onset of the event rather than the delayed threshold crossing.

***Stage 2: Phase classification:*** Once a phase had been detected, it was classified by the dual-branch network, which combined a learned representation of the raw signal with the 17 handcrafted features. The convolutional branch learned the temporal waveform pattern directly from the signal, whereas the feature branch supplied amplitude-invariant descriptors that remained stable across the gain and electrode differences between the public training dataset and the in-house wearable hardware, so that the network drew on both data-driven and prior knowledge and could therefore operate across the two domains. The input to the convolutional branch was the central 270-sample window of the detected phase, which passed through a stack of 1D convolutional blocks and a GAP pooling layer to produce a fixed-length signal embedding. At the same time, the 17 handcrafted features computed over the phase passed through a small fully connected branch, as shown in Figure 3, to produce a feature embedding. The two embeddings were then concatenated and passed through a final fully connected layer and a softmax that produced the phase label (classification).

### 2.4. Firmware Export and On-Device Inference

Both networks were developed and trained offline, but they had to run on the microcontroller, which has no DL runtime. Therefore, each trained network was exported as portable C code, in the form of a header file that contains the trained weights together with a light inference routine, and this code was compiled directly into the firmware. Exporting the model using this approach, rather than relying on an interpreter or an external library, keeps the inference self-contained and allows it to run within the time and memory budget of the device. To reduce the computation for each inference, the batch-normalization parameters of every layer were folded into the weights and biases of the preceding convolutional or fully connected layer. Because batch normalization reduces to a fixed affine transformation once training is complete, it can be merged with the preceding linear operation without altering the output, so that each layer becomes a single weight-and-bias operation followed by its activation. The processing carried out on the device reproduced exactly the operations used during the training stage: the signal window is z-scored per window, and the handcrafted features are normalized using the statistics estimated on the training set. Consequently, the input presented to the embedded network is identical in form to the input it was trained on. Because the embedded inference path was re-implemented in C rather than executed by the framework used for training, it was essential to verify its numerical equivalence to the trained model before evaluation. Hence, the C implementation and the reference implementation were run on the same held-out data, and the two produced identical class decisions on every window, with only negligible differences in the output probabilities attributable to floating point rounding.

### 2.5. Real-Time Output Evaluation

***Participants:*** Thirty healthy adults with the characteristics shown in Table 2 took part in the study. Inclusion criteria were: aged between 18 and 45 years, able to perform STS and StS movements independently without using the arms or any assistive device, and free from current lower limb pain. Exclusion criteria were: previous knee injury or surgery; diagnosed neuromuscular or musculoskeletal disorder affecting the lower limbs; cardiovascular condition contraindicating moderate physical exertion; and pregnancy. The study was approved by the Faculty of Engineering and Physical Sciences Research Ethics Committee at the University of Leeds, United Kingdom (approval LTELEC-004). Each participant received an information sheet and gave written informed consent prior to the experiment day. On the experiment day, each participant was familiarized with the task before the actual recording began.

**Figure 4:**
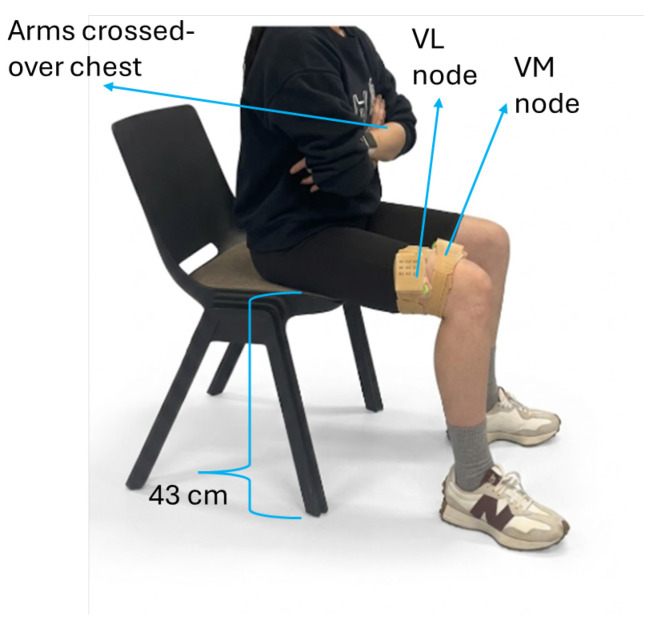
Experimental setup showing the sensing node placement on the vastus lateralis (VL) and vastus medialis (VM) of the right quadriceps, with the participant in the arms-crossed seated start position on a 43 cm armless chair.

***Experimental Protocol:*** The developed in-house sEMG sensing system was used to record the signals from the VL and VM of the right leg, consistent with the side recorded in the Gait120 training dataset. Prior to placement of the sensing nodes, the VM and VL sites were shaved using a disposable razor, then alcohol wipes were used to clean the area. Each subject was then asked to perform repeated STS and StS movements. Participants sat on a standard armless chair of fixed height (≈43 cm), following the chair-stand test [20], with arms crossed over the chest and feet placed at fixed positions marked on the floor to keep each participant’s foot placement consistent across the experimental sessions. The experimental setup is shown in Figure 4. The subjects were asked to perform the movements at a self-selected pace for a continuous one minute. This protocol was repeated on the same day for three sessions, with a 30 min rest period between sessions. At the start of each session, the participants remained seated for 3 s, during which the sensing system was calibrated.

***Repeated-Session Validation of the Deployed Model:*** To assess the stability of the model across repeated use on the same day, each subject performed three sessions, each separated by a 30 min seated rest. The sensing nodes were left in place on the skin between sessions, and no retraining or recalibration was performed. The model was then evaluated independently on each session, and for every session the detection rate and the phase-classification accuracy were computed per subject. The results were reported as mean ± standard deviation (SD) across subjects for each session. Differences between sessions were tested with the Wilcoxon signed-rank test, with significance at p<0.05 [41]. Because the sensing nodes remained attached throughout, this analysis characterizes robustness to repeated same-day use and session-to-session variability rather than to sensor removal and re-placement.

***Ground Truth and Evaluation:*** At this stage, two aspects of the system were evaluated: the classification of each phase, and the detection of its onset/offset. Each session began with the participant seated; thus, the repeated transitions followed a fixed alternating order. Consequently, the correct label of each phase was known from its position in this order, and these known labels served as the reference for evaluation. The system classified each phase in real time without any knowledge of the order, and its predictions were then compared against the reference label. The detection was evaluated by applying the approximated generalized likelihood ratio (GLR) offline detector [21] to the recorded sEMG signals. Being non-causal and operating on the entire signal, this offline detector served as the timing reference for both the count of true transitions and the timing of the onset/offset boundaries (accuracy).

***Ablation of the Dual-Branch Design:*** To assess the contribution of each branch and determine whether the added computational cost of the dual-branch design was justified, the full classifier was compared with two simplified versions. The first used only the 1D-CNN signal branch with the 270-sample VL/VM window, while the second used only the fully connected branch with the 17 handcrafted features. All three models were evaluated using the same data windows, pre-processing steps, training settings, and leave-one-subject-out protocol, so the only difference was the input branch used. For each model, classification accuracy was reported as mean ± SD across subjects, together with the embedded computational cost in terms of parameter count, multiply–accumulate operations, and memory usage. The improvement achieved by the dual-branch model over each single-branch model was assessed using a paired Wilcoxon signed-rank test [41], with significance set at p<0.05.

***Metrics:*** Classification performance was reported as the overall accuracy with the precision, recall, and F1−score of each class, together computed from the confusion matrix following the standard classification metrics [42]:

(4) $$\mathrm{Precision}=\frac{TP}{TP+FP}$$

(5) $$\mathrm{Recall}=\frac{TP}{TP+FN}$$

(6) $$F1-score=\frac{2\cdot \mathrm{Precision}\cdot \mathrm{Recall}}{\mathrm{Precision}+\mathrm{Recall}}$$

(7) $$\mathrm{Accuracy}=\frac{\sum_{i}TP_{i}}{N}$$

For both the offline and real-time evaluations, the classifier used 270-sample windows, with performance assessed at the phase level using one prediction from the central 270-sample window of each phase. The same approach was applied to the offline Gait120 test set and the real-time wearable recordings. Classification performance was reported as the mean ± standard deviation across participants, together with class-specific precision, recall, and F1-score, as well as the macro-averaged F1-score. Generalization to unseen participants was evaluated using leave-one-subject-out cross-validation, with results averaged across folds. Detection performance was assessed using the detection rate, defined as the proportion of completed phases correctly detected, together with the false detection rate. Detection timing was evaluated by comparing the detected onset and offset with the offline reference and reporting the mean ± standard deviation and root mean square error. To examine stability across repeated sessions, classification accuracy and detection rate were also reported separately for each session and compared between sessions. Differences between model configurations and sessions were evaluated using the paired Wilcoxon signed-rank test, with statistical significance set at p<0.05. End-to-end latency was defined as the time between the end of a phase and the availability of the classification result and was estimated from the processing pipeline, including both the offset confirmation delay introduced by the stabilizer and the time required for feature extraction and classification.

### 2.6. Benchmarking Against Existing Methods

The most relevant previous methods report their performance using different datasets, numbers of channels, sensing modalities, and offline processing environments. Because each of these factors affects the difficulty of the task, directly comparing their reported accuracies would not provide a fair assessment. Representative methods were therefore re-implemented and evaluated under the same conditions as the proposed approach. All methods used the same data and evaluation metrics and were tested on both the public Gait120 dataset and the real-time wearable recordings. The classification models were evaluated using the same leave-one-subject-out (LOSO) folds as the proposed classifier. Similarly, the detection baselines were applied to the same recordings and evaluated against the same offline reference. This ensured that the observed differences were primarily due to the models rather than differences in the experimental setup.

For classification, the proposed dual-branch classifier was compared with two groups of baseline models. The first group consisted of conventional feature-based classifiers: linear discriminant analysis (LDA), support vector machine (SVM), weighted k-nearest neighbors (wKNN), and random forest (RF) [26,27,39]. These models represent the established lightweight approach to sEMG pattern recognition and were trained using the same 17 handcrafted features provided to the feature branch of the proposed model. The second group consisted of deep sequence models: a temporal convolutional network (TCN) [29] and a CNN-BiLSTM [31]. The TCN was selected as a compact model capable of learning long-range temporal patterns while remaining suitable for embedded deployment. The CNN-BiLSTM was included as a higher-capacity model that combines convolutional feature extraction with bidirectional recurrent modelling. Both models were trained using the same 270-sample VL and VM windows provided to the signal branch.

For movement detection, the proposed per-burst CNN detector was compared with both classical and learned onset/offset detection methods. The classical methods included the Teager–Kaiser energy operator (TKEO) [22], the double-threshold detector [19], and the approximated generalized likelihood ratio (AGLR) [21]. These methods were selected because they represent three widely established approaches in the sEMG literature, based respectively on energy operators, statistical thresholds, and likelihood ratio testing. The learned methods included the neural muscle activation detector (NMAD) [23], an LSTM-based muscle activation detector (LSTM-MAD), and a TCN-based detector [29]. Together, these models represent the learned detection family: NMAD provides a published recurrent benchmark, while LSTM-MAD and the TCN detector represent recurrent and temporal-convolutional alternatives.

Both classification and detection were evaluated using the same metrics as the proposed model. Classification performance was assessed using accuracy and class-specific precision, recall, and F1-score. Detection performance was assessed using the detection rate, false detection rate, and onset and offset timing errors. The embedded computational cost of each model was also reported in terms of parameter count, memory footprint, and inference latency. Differences between each baseline and the proposed model were evaluated using the paired Wilcoxon signed-rank test [41], with statistical significance defined as p<0.05.

## 3. Results

This section presents the performance of the system in two stages. The model’s behavior was first established offline on the Gait120 dataset under leave-one-subject-out cross-validation, and its numerical equivalence after deployment to the device was verified. The system was then evaluated in real-time on the 30 participants, covering the classification of each phase, the detection of phase onset/offset, and the end-to-end latency of the on-device pipeline.

### 3.1. Offline Model Performance

***Classification:*** Table 3 reports the dual-branch classifier under leave-one-subject-out cross-validation. On the held-out folds of Gait120, the network distinguished STS from StS movements with an overall accuracy of 99.6% (mean 99.7 ± 1.9% across subjects). The performance was balanced across the two classes: STS had a precision of 100%, recall of 99.3%, and an F1-score of 99.6%, while StS reached a precision of 99.3%, recall of 100%, and an F1-score of 99.6%. Of the 558 STS and 569 StS phases, only 4 STS phases were misclassified and all StS phases were correctly classified.

***Onset/Offset detection:** *Table 4 summarizes the detection performance of the per-burst classifier on the Gait120 dataset. Of the 176 labeled movement phases, 171 were detected and 170 were matched to the labeled phase, giving a detection rate of 96.6% and a false detection rate of 0.6%. Measured against the annotated onset/offset boundaries, the onset error was 30 ± 217 ms and the offset error was 2 ± 283 ms.

### 3.2. Real-Time Evaluation

***Classification:*** Table 3 reports the classification performance of the dual-branch network on the real-time wearable sensing system. The trained model was evaluated across 30 healthy subjects who performed 829 movement phases (415 STS, 414 StS); the network classified STS and StS with an overall accuracy of 85.6% (mean 80.6 ± 15.3% across subjects). STS was classified with a precision of 84.4%, a recall of 87.5%, and an F1-score of 85.9%. On the other hand, StS showed a precision of 87.0%, a recall of 83.8%, and an F1-score of 85.4%. Of the 415 STS and 414 StS phases, 52 and 67 were misclassified, respectively.

***Onset/Offset detection:*** Table 4 shows the detection performance on the wearable sensing system. Of the 1787 labeled movement phases across the 30 subjects, the onset/offset detection rate was 82.0% and the false detection rate was 9.7%. The onset error at run-time was −49.8 ± 167.0 ms; the negative sign indicates that the detector flagged the onset slightly before the reference. On the other hand, the offset error was 66.9 ± 143.4 ms; the positive sign of the offset error indicates that the offset was flagged slightly after.

***Repeated-Session Validation of the Deployed Model:*** Table 5 summarizes the model’s performance across repeated same-day sessions using the wearable data. Each participant completed up to three sessions, and the model was evaluated separately for each session. The classification accuracy was 80.2±16.7% in Session 1, 78.7±20.5% in Session 2, and 85.3±18.7% in Session 3. The corresponding detection rates were 87.9±15.0%, 89.9±14.5%, and 86.9±19.7%, respectively. Although some variation was observed across sessions, the Wilcoxon signed-rank test found no significant pairwise differences in classification accuracy (S1–S2: p=0.31; S1–S3: p=1.00; S2–S3: p=0.25) or detection rate (S1–S2: p=0.22; S1–S3: p=0.09; S2–S3: p=0.25), with all comparisons showing p>0.05. The per-session rates are averaged across subjects, whereas the overall detection and classification rates are pooled across all movements and phases; the two aggregations can differ when a few subjects contribute many movements.

Table 6 summarizes the computational cost and execution frequency of the network models. The per-burst detector requires ≈0.61 million multiply–accumulate operations per window and ≈6200 parameters, while the dual-branch classifier requires ≈1.54 million operations and ≈152,000 parameters. Stored as 32-bit floating point weights, the detector occupies ≈24 KB and the classifier ≈594 KB, a combined footprint of ≈618 KB; the detector runs on each sensing node within its on-chip memory, while the larger classifier is held in the external PSRAM on the hub. The detector, which runs once per 90 ms window, is the steady-state load; the classifier runs only once per completed movement.

The end-to-end latency, from the end of a phase to the availability of its classification result, was characterized analytically as the sum of the offset confirmation delay imposed by the stabilizer and the computational cost of one feature extraction and classification pass. The stabilizer confirms the end of a phase after a single below-threshold burst (90 ms), and one classification pass (≈1.54 million multiply–accumulate operations, ≈152,000 parameters) completes in under 10 ms on the 240 MHz processor. The end-to-end latency was therefore ≈100 ms, dominated by the offset confirmation delay.

***Ablation of the dual-branch design:*** Table 7 shows the contribution of each branch under the same leave-one-subject-out evaluation protocol. The complete dual-branch classifier achieved an accuracy of 81.4±15.1%, compared with 77.7±20.0% for the signal branch alone and 67.2±17.6% for the feature branch alone. The improvement provided by the dual-branch design was statistically significant relative to both individual branches (dual versus signal-only, p=0.038; dual versus feature-only, p=0.0038; Wilcoxon signed-rank test). Importantly, the gain over the signal-only model required only a modest increase in computational cost, from 146,530 to 151,970 parameters and from 572 to 594 KB of memory. Although the feature-only model was considerably smaller, with 5442 parameters and a 21 KB memory footprint, its accuracy was substantially lower. The dual-branch architecture was therefore retained because it provided a meaningful improvement in accuracy at a small additional cost, while remaining suitable for deployment on the ESP32-S3.

***Benchmark against existing methods:*** Table 8 compares the classification and detection methods under the same leave-one-subject-out protocol on both datasets and also reports the parameter count, memory footprint, and inference time of each model. For classification, the proposed dual-branch model achieved 99.6% accuracy on Gait120 and 85.6% on the wearable recordings. Its performance on Gait120 was comparable to that of the TCN (99.6%, p=0.66) and CNN-BiLSTM (99.5%, p=0.63), with no significant differences between them. However, it significantly outperformed all feature-based classifiers, whose accuracies ranged from 83.8% to 91.3% (all p<0.001). On the more challenging wearable recordings, the proposed model significantly outperformed every baseline, including the CNN-BiLSTM (78.9%, p=0.032) and the TCN (69.8%, p=0.001). It required approximately 152,000 parameters and 594 KB of memory, compared with approximately 335,000 parameters and 1309 KB for the CNN-BiLSTM and approximately 22,000 parameters and 88 KB for the TCN.

For detection, the proposed per-burst model achieved a detection rate of 97.1% on Gait120 and 82.0% on the wearable recordings, with a false detection rate of 9.7%. On the wearable data, the TCN achieved 92.8% (p=0.85), while LSTM-MAD and NMAD achieved 81.6% (p=0.012) and 77.5% (p=0.003), respectively. The classical methods performed substantially worse, with detection rates between 18.6% and 23.9% for TKEO, the double-threshold detector, and AGLR (all p<0.001). Despite its compact size, the proposed detector required only approximately 6200 parameters and 24 KB of memory.

## 4. Discussion

The present study proposed and evaluated a novel method for detecting the onset/offset of sit-to-stand (STS) and stand-to-sit (StS) transitions. The approach relied on an in-house wearable sEMG sensing system using two channels (VL and VM) with a per-burst convolutional detector, and a dual-branch convolutional classifier, deployed on the device to operate in real-time. The results demonstrated that both tasks are feasible on the wearable sEMG sensing system in real-time: the movements were detected reliably and with few false detections. This low false detection rate was achieved through the design of the per-burst detector, which combines exponential smoothing of the per-burst probabilities, dual-threshold hysteresis on the smoothed probability, and a persistence requirement to enforce sustained activation across consecutive bursts before declaring an event. Consequently, brief probability rises from noise or partial activations are not reported as detection events. The complete pipeline operated within a latency short enough for interactive use on the embedded processor. Beyond establishing feasibility, both tasks fell from their offline benchmark by a similar margin on the wearable (detection from 96.6% to 82.0%; classification from 99.6% to 85.6%), indicating that the move from curated recordings to on-body acquisition imposes a comparable cost on each. The residual gap reflects what each task demands of the underlying signal. Localizing a movement requires only that the activation burst be resolved in time, which remains comparatively robust to the wearer’s recording conditions and inter-subject variability. Discriminating which movement occurred depends instead on the finer temporal structure of the activation envelope, namely the concentric build-up of the STS against the eccentric, front-loaded braking of the StS. Together with the limited direction information available from two co-active knee extensor channels, this finer structure sets the ceiling on identification performance.

***Movements classification:*** The movements classification on the wearable sensing system achieved a lower accuracy than the offline result on the public dataset, while remaining effective, indicating that the classification holds when the model is applied in the deployed setting and not only on the development data. The two evaluations reported in Table 3 are not directly equivalent, as the offline value was obtained on the curated dataset, while the wearable sensing system value was obtained under on-body acquisition. However, the classification was comparable for both movements, with similar precision and recall for STS and StS. Because the movements were performed in an alternating sequence, this balance indicates that the classifier discriminates them by the characteristics of the movements rather than their order or relative frequency, which is the behaviour required for dependable use in practice, such as during a rehabilitation session. The difference shown in Table 3 between the wearable and offline accuracy is consistent with the change from the curated dataset to on-body acquisition on off-the-shelf hardware. On the wearable, accuracy also varied far more across subjects than offline (80.6 ± 15.3% versus 99.7 ± 1.9%), indicating that on-body acquisition affects some participants more than others. This spread is consistent with per-subject differences in skin–electrode contact, soft-tissue thickness, and movement execution that the curated dataset does not contain. The pooled accuracy (85.6%) sits above the subject mean (80.6%) because participants who contributed more transitions were, on average, classified more reliably. Reducing this between-subject variability, for example through a short per-user calibration, is therefore the most direct route to improving real-time performance. Moreover, the per-class recall was balanced in both evaluations, with no consistent bias between the two movements. Offline, StS was recovered marginally better than STS (100% versus 99.3% recall), whereas on the wearable the order reversed (STS 87.5% versus StS 83.8%). The two transitions are governed by different contraction modes, with STS driven by the concentric knee extensor effort that raises the body and StS by the eccentric activation that controls the descent [1]; however, this physiological difference did not translate into a systematic recall asymmetry, since the direction of the small difference changed between the two settings. It is therefore most plausibly attributed to subject-level variability rather than to a property of either movement, and it does not disturb the overall balance between the two classes. The VL and VM are synergist knee extensors that are co-active in both transitions, so their activation overlaps across the two movements. Because of this overlap, the phases are separated not by which muscle is active or by activation level, but by the timing and shape of the shared activation. Surface EMG from this pair therefore distinguishes the phases through this temporal structure, which the 1D-CNN signal branch learns from the activation window.

***Onset/offset detection:*** The onset/offset detection results shown in Table 4 showed a lower detection rate (82.0% on the wearable vs. 96.6% offline) and a higher false detection rate (9.7% on the wearable vs. 0.6% offline). This difference arises because the deployed model on the wearable sensing system handles continuous on-body recording, which removes the clean separation between successive movements present in the offline dataset; the missed and false detections therefore concentrate at the junctions between adjacent phases, where the activation does not fully subside and the boundary between one movement and the next is genuinely indistinct. The higher false detection rate follows from the finer temporal resolution the detector applies to separate the rapid, self-paced transitions of continuous on-body recording. Where the offline curated data spaced each movement apart, the wearable protocol places successive transitions within a fraction of a second of each other, so the detector must resolve closely spaced boundaries, and at these junctions it occasionally commits an additional detection. These false detections therefore arise at the same indistinct boundaries as the missed ones, and represent the cost of resolving back-to-back movements rather than a failure to model the activity, since a clean separation does not exist in continuous use. At such junctions the boundary is not expressed in the muscle activity, hence it cannot be recovered from the two-channel sEMG on which the present system relies.

The timing errors in Table 4 are not symmetric, and the sign of each is informative. The asymmetric signs of the onset and offset errors arise from the stabilizer’s design, in which the persistence requirement delays the declaration of either boundary, while the look-back buffer is applied at the onset to undo that delay. Together, they place the detected onset just ahead of the true start and the detected offset just beyond the true end; consequently, the captured window brackets the movement with a small margin at both ends, so the full activation is retained rather than truncated. The only consequence of this outward bias is that a phase duration computed from the two boundaries is slightly overestimated; this is immaterial for triggering, but would need to be reconciled before the boundaries are used to measure movement duration.

Table 4 shows that the spread of the timing error (its standard deviation) is larger than its mean. This variation does not reflect an error in the detector. Rather, it arises because the detector assigns a state to each burst as a whole, while the exact start and end of a continuous movement naturally vary between repetitions and individuals, even under ideal measurement conditions. The small mean errors therefore indicate that the detector localizes the boundaries accurately on average, with the spread reflecting the natural variability of the transitions themselves. The present study is a proof-of-concept study scoped to the sit-to-stand and stand-to-sit task rather than to free-living, multi-activity monitoring, and this scope shapes how the system would behave around other daily activities such as walking or turning. Surface EMG is strongly task-dependent: different movements elicit different muscle-activation patterns, so a model trained on one task encodes representations specific to it. This task-dependence has been shown for the knee extensors, where VM sEMG alone can separate distinct lower limb tasks such as standing, sitting, and walking [43]. Because the present classifier was trained only on STS and StS phases and has no rejection class, in an unconstrained setting a sustained knee extensor contraction from another activity, for example the loading phase of walking or a turn, could be detected by the per-burst detector and then forced into one of the two transition labels, producing a false trigger. The detector’s stabilization stage (exponential smoothing, hysteresis, and persistence) suppresses brief and transient activity, but it does not suppress sustained non-task contractions, so it cannot reject such activities on its own. Handling the integration of other daily activities would therefore require an explicit activity-gating front-end, or an added rejection class that first decides whether a movement is an STS/StS transition before classifying its direction. This is a necessary step for extending the present feasibility study toward free-living use, and is left to future work.

The repeated-session results in Table 5 show that the deployed model maintained a similar level of performance across the same-day sessions. Neither classification accuracy nor detection rate differed significantly between any pair of sessions (Wilcoxon signed-rank test, all p>0.05). This stability may be partly explained by the use of amplitude-invariant features and per-channel normalization, which were designed to reduce the effect of changes in signal gain and baseline drift while the electrodes remained attached to the skin. As all sessions were completed on the same day without removing or repositioning the sensor nodes, these findings reflect short-term session-to-session consistency during continued wear rather than robustness to electrode re-placement or use on different days. Within this setting, the lack of a systematic decline suggests that the model can maintain its performance over a continuous wearing period.

The present system uses off-the-shelf MyoWare 2.0 sensors, which differ from the laboratory-grade equipment used to collect the Gait120 training data. Because these sensors use a compact single-differential electrode arrangement with on-board amplification, they generally provide a lower signal-to-noise ratio and therefore record more baseline noise than research-grade systems [44,45]. Their signals are also more affected by changes at the skin–electrode interface. As the electrode gel dries and perspiration builds up during wear, the baseline can drift, and the effective signal gain can vary both within and between sessions [44]. Furthermore, mounting the sensor on the moving thigh makes the signal more vulnerable to low-frequency motion artifacts caused by movement of the skin, soft tissue, and cabling, some of which may overlap with the sEMG frequency range. However, these effects are reduced rather than completely removed in the current pipeline. The implemented band-pass and notch filters suppressed out-of-band noise and mains interference, while the amplitude-invariant features and per-channel normalization reduce the effect of baseline and gain differences. Despite the limitations of the off-the-shelf hardware, this processing approach maintained real-time performance at a practical level. This supports the feasibility of using an off-the-shelf wearable system for direction-aware STS and StS detection without relying on laboratory-grade acquisition equipment.

***Real-time feasibility:*** Referring to Table 6, the DD-CNN is compact: the per-burst detector requires 0.61 million MACs and ≈6200 parameters, the dual-branch classifier requires ≈1.54 million MACs and ≈152,000 parameters, and the combined memory footprint is ≈618 KB, which exceeds the 512 KB on-chip SRAM and is therefore held in the ESP32-S3’s external PSRAM on the hub. With the detector operating on each burst and the classifier once per detected movement, the inference cost is negligible relative to the available processing interval. Moreover, the end-to-end latency is dominated by the stabilizer’s offset confirmation requirement rather than by the hardware. As this latency was characterized analytically from the pipeline, it represents the expected responsiveness of the deployed system.

***Significance of the dual-branch design:*** The ablation results in Table 7 show that the two branches make distinct but complementary contributions. The signal branch provided most of the discriminative power, achieving 77.7% accuracy on its own. This indicates that the 1D-CNN learned most of the direction-specific waveform patterns directly from the two sEMG channels. In comparison, the feature branch alone achieved 67.2%, showing that the seventeen handcrafted features contained useful directional information but were insufficient to reliably distinguish the two transitions by themselves; the main benefit of the feature branch, however, was not limited to the increase in mean accuracy. Adding it to the signal branch improved accuracy from 77.7% to 81.4% (p=0.038), while reducing the between-subject standard deviation from 20.0 to 15.1 percentage points. The handcrafted features therefore made the classifier more consistent across individuals, particularly for participants on whom the raw signal model performed poorly. This behavior is consistent with the use of amplitude-invariant descriptors, which are less sensitive to differences in signal gain, electrode placement, and acquisition conditions between the public dataset and the in-house wearable recordings. They therefore provide a more stable representation when variations in raw signal scaling might otherwise affect the prediction. This improvement was achieved at little additional computational cost. The feature branch contributed only approximately 5440 of the model’s 151,970 parameters, corresponding to about 3.6% of the total model and approximately 22 KB of memory. The convolutional signal branch therefore remained the main source of computational cost. Overall, the dual-branch design is justified not only by its improvement in average accuracy, but more importantly by the greater cross-subject and cross-domain consistency it provides at minimal additional cost. This is particularly important for a model trained on a public dataset and deployed without subject-specific retraining on wearable hardware.

***Our work vs. existing methods:*** The benchmark in Table 8 compares the proposed model with representative classical and deep approaches on both datasets. On the curated Gait120 data the proposed model, the TCN, and the CNN-BiLSTM all saturated near 99.6% and were statistically indistinguishable, while the classical feature-based classifiers trailed by eight to sixteen points, so clean, well-separated recordings do not discriminate between the stronger models, whereas the wearable recordings did. There the proposed model was the most accurate (85.6%) and more consistent across subjects than the CNN-BiLSTM (80.6±15.3% versus 71.7±20.0% mean), while the TCN was lower still (64.6±13.7%). The pure end-to-end baselines learn from the raw waveform and have no defence against the change in gain, electrode placement, and baseline that separates the curated dataset from on-body acquisition, so they fit the clean distribution well but degrade and become erratic once it shifts. The proposed model resists this shift through its amplitude-invariant feature branch, the same mechanism that lowered the between-subject standard deviation in the ablation. Its advantage is therefore not capacity, which the deep baselines match on clean data, but built-in robustness to the deployment domain shift, achieved at roughly half the size of the CNN-BiLSTM.

The detection benchmark separates the methods along a different axis. The classical detectors (TKEO, double-threshold, and AGLR) reached about 95% offline but fell below 25% on the wearable, because each declares activity by comparing a statistic to a threshold estimated from a rest baseline, and in continuous, back-to-back transitions that quiet baseline never occurs, so the baseline-derived threshold is unreliable and most boundaries are missed. The learned detectors avoid this failure because they recognize the shape of an activation burst rather than its level relative to rest, and all of them, including the proposed detector, remained usable on the wearable. Among the learned detectors the comparison is best read as a balance between detection rate and false detections rather than detection rate alone. The proposed detector held a low false detection rate (9.7%), whereas NMAD and LSTM-MAD, at similar or lower detection rates, produced markedly more false detections (18.1% and 13.5%), a difference attributable to the smoothing, hysteresis, and persistence stages that suppress spurious activity. NMAD’s reported strength, precise onset timing, was established on simulated signals with exact activation labels and on a single runner under controlled conditions, where bursts are clean and well separated, so it does not transfer directly to the continuous, self-paced, multi-subject recordings used here. As a recurrent network, NMAD was also the largest learned detector in the benchmark, about 303 KB against 24 KB for the proposed detector, and it was not demonstrated on-device. The TCN detector reached a higher detection rate at a comparable false detection rate, with no significant difference from the proposed detector (p=0.85). Detection, however, is the easier of the two tasks, and the decisive comparison is the complete system, which must also discriminate direction; there the same TCN was significantly less accurate than the proposed classifier on the wearable (69.8% versus 85.6%, p=0.001), so a TCN-based system would localize movements adequately but fail to identify their direction reliably.

The cost columns of Table 8 separate the methods more sharply than the accuracy columns. Accuracy alone does not determine which model can run on the wearable. The CNN-BiLSTM, the only baseline that approached the proposed model on the wearable, occupies about 1.31 MB, which exceeds the 512 KB on-chip memory of the ESP32-S3, and its bidirectional recurrence requires the complete sequence before a decision, both of which complicate streaming deployment. The classical feature-based classifiers are small but inaccurate, and the random forest is both the largest classifier (about 2.36 MB) and the slowest (about 224 ms per inference), making it impractical on the device. Among the detectors, most learned models are small enough for the device; NMAD is the exception, the heaviest at about 303 KB and never run on-device, whereas the proposed detector is among the smallest at 24 KB. The proposed networks fit within the device budget, a 618 KB combined footprint held in PSRAM, execute well inside the available interval, and are the only models in the comparison that were exported, parity-verified, and measured on the hardware. The benchmark therefore separates the methods on two axes at once, accuracy under on-body noise and the cost of running on a commodity microcontroller, and the proposed system is the only one competitive on both. Across both tasks the same theme emerges. The proposed system is not the highest-capacity model and does not win on clean, curated data, where several baselines are its equal. It wins where deployment actually happens, on continuous, on-body recordings from off-the-shelf hardware, and it does so through design choices aimed squarely at that setting, namely the amplitude-invariant feature branch that anchors classification against per-subject signal scaling and the stabilized per-burst detector that holds false detections down without a rest baseline. Its distinguishing contribution is therefore the regime in which it operates, performing onset/offset detection and direction discrimination together, from only two knee extensor sEMG channels, in real-time on the device, with the deployed computational cost quantified rather than assumed, a combination that none of the compared methods provides.

***Future Work:*** The present study is a proof-of-concept evaluated exclusively in healthy adults. The clinical applications that motivate it, namely rehabilitation, assistive-device control, and fall-risk monitoring, were not tested, so the system’s performance in clinical populations remains to be established. Future work will therefore extend the evaluation to clinical populations, such as people with knee osteoarthritis or those undergoing lower limb rehabilitation, in whom altered activation and movement patterns may affect both detection and identification. The identification stage will be examined with additional muscle channels whose role differs between rising and lowering, such as antagonist or postural muscles, to determine whether movement direction can be separated beyond the level attainable from the knee extensors alone. The detection stage will be extended to handle the boundary ambiguity at the junction between consecutive self-paced transitions, where the absence of a clean rest gap was one source of the missed and false detections observed here. The analytically characterized latency will be confirmed by direct on-device measurement of the inference time and end-to-end delay under continuous operation, complementing the computational analysis presented in this study. The present study was designed as an initial controlled validation of the proposed approach under standardized sensor placement; cross-session testing after sensor removal and re-placement represents a separate reproducibility stage and will be addressed in subsequent validation work. In that stage, several measures could reduce the risk from re-placement. The amplitude-invariant features and per-channel normalization already used in the pipeline absorb much of the change in signal gain and baseline that re-placement introduces, and this robustness could be strengthened by placing the electrodes over fixed anatomical landmarks with consistent skin preparation and by a short calibration at the start of each session to re-estimate the normalization after the sensors are repositioned.

## 5. Conclusions

This study presented a method that performs onset/offset detection and direction discrimination of sit-to-stand and stand-to-sit movements in real-time on an in-house, two-channel sEMG sensing system built from off-the-shelf hardware. The system couples a per-burst convolutional detector, stabilized through exponential smoothing, hysteresis, and persistence, with a dual-branch convolutional classifier that combines the raw two-channel signal with seventeen handcrafted, amplitude-invariant features. Trained offline on the public Gait120 dataset and deployed unchanged with firmware parity to the reference model, the system reached 99.6% classification accuracy and 96.6% detection completeness on held-out public data, and retained 85.6% classification and 82.0% detection in real-time across thirty healthy adults, within ≈100 ms latency and a 618 KB footprint. Under a common leave-one-subject-out benchmark, the classifier matched the strongest deep baselines on the public data and outperformed them on the noisier wearable recordings while remaining markedly smaller, and the detector remained robust on continuous on-body recordings where classical detectors failed. Demonstrated here in healthy adults, the approach indicates potential applications such as real-time movement monitoring, lower limb rehabilitation, and assistive-device control, with validation in clinical populations left to future work.

## Figures and Tables

**Table 1:** Computed handcrafted features.

Feature	Channel	Description	Family
Peak position	VL, VM	Temporal position of the mean-absolute-value (MAV) envelope peak, normalized to the phase length	Envelope-shape
Rate of EMG rise (RER)	VL, VM	Initial slope of the MAV envelope, normalized by the peak MAV	Envelope-shape
VM/VL ratio	—	Ratio of mean MAV of VM to mean MAV of VL	Envelope-shape
Waveform length (WL)	VL, VM	Mean absolute sample-to-sample difference, normalized by the mean absolute value	Temporal [39]
Zero-crossing rate (ZCR)	VL, VM	Rate of sign changes of the signal	Temporal [39]
Slope-sign-change rate (SSC)	VL, VM	Rate of sign changes of the first difference	Temporal [39]
Median frequency (MDF)	VL, VM	Median of the power spectral density (Welch estimate)	Spectral [27]
Hjorth mobility	VL, VM	$\sqrt{\mathrm{var}\left(x^{\prime }\right)/\mathrm{var}\left(x\right)}$	Complexity [40]
Hjorth complexity	VL, VM	Mobility$\left(x^{\prime }\right)$/Mobility(x)	Complexity [40]

**Table 2:** Participant characteristics. Age, mass, height, and body mass index are reported as (mean ± standard deviation).

Characteristic	Value
Participants (n)	30
Gender (male:female)	16:14
Age (years)	27.38 ± 4.55
Height (cm)	169.72 ± 9.88
Weight (kg)	69.38 ± 12.68
Body mass index (kg/m^2^)	24.38 ± 2.97

**Table 3:** Phase-classification performance for the offline Gait120 evaluation and the real-time wearable recordings, both under leave-one-subject-out (LOSO). Values are percentages.

Evaluation	Movement	Precision	Recall	F1-Score	Accuracy (Pooled)	Accuracy (Mean ± SD)
Offline—Gait120	STS	100	99.3	99.6	99.6	99.7 ± 1.9
	StS	99.3	100	99.6		
Real-time—wearable	STS	84.4	87.5	85.9	85.6	80.6 ± 15.3
	StS	87.0	83.8	85.4		

**Table 4:** Offline detection performance on the Gait120 dataset and real-time wearable sensing system.

Metric	Offline Model Value	Real-Time Value
True movements	176	1787
Detection rate	96.6%	82.0%
False detection rate	0.6%	9.7%
Onset error (mean ± SD)	30 ± 217 ms	−49.8 ± 167.0 ms
Offset error (mean ± SD)	2 ± 283 ms	66.9 ± 143.4 ms

**Table 5:** Repeated-session performance of the deployed model on the wearable data. Values are mean ± SD across subjects; *p*-values from the Wilcoxon signed-rank test, S #: Session number.

Metric	Session (Mean ± SD, %)			Wilcoxon *p*		
	S1	S2	S3	S1–S2	S1–S3	S2–S3
Classification accuracy	80.2±16.7	78.7±20.5	85.3±18.7	0.31	1.00	0.25
Detection rate	87.9±15.0	89.9±14.5	86.9±19.7	0.22	0.09	0.25

**Table 6:** Computational complexity, memory footprint, and execution frequency of the deployed network models.

Network	Runs	MACs per Inference	Parameters	Memory
Detector (per-burst active/rest)	per window (∼90 ms)	0.61 M	∼6.2 k	∼24 KB
Classifier (dual-branch)	per movement (∼1–2 s)	1.54 M	∼152 k	∼594 KB

**Table 7:** Ablation of the dual-branch classifier under leave-one-subject-out. Accuracy is mean ± SD across subjects; *p*-values are from the paired Wilcoxon signed-rank test versus the full dual-branch model.

Configuration	Accuracy (%)	*p* vs. Dual	Parameters	Memory
Dual-branch (full)	81.4±15.1	—	151,970	594 KB
Signal branch only (1D-CNN)	77.7±20.0	0.038	146,530	572 KB
Feature branch only (FC)	67.2±17.6	0.0038	5442	21 KB

**Table 8:** Benchmark against existing methods under leave-one-subject-out on the Gait120 and wearable datasets. Classification is reported as accuracy and detection as detection rate; *p*-values are from the paired Wilcoxon signed-rank test versus the proposed model on the wearable data. Parameters, memory, and inference time give the embedded cost of each model; inference time is the per-inference compute time measured on the benchmarking workstation, and is distinct from the on-device end-to-end latency.

Model/Method	Gait120 (%)	Wearable (%)	*p* (Wear.)	Params	Mem. (KB)	Inf. Time (ms)
*Classification (accuracy)*						
Proposed (dual-branch)	99.6	85.6	—	151,970	594	16.8
CNN-BiLSTM	99.5	78.9	0.032	335,074	1309	7.8
TCN	99.6	69.8	0.001	22,402	88	4.9
Random forest	91.3	64.4	<0.01	n/a	2357	224
Support vector machine	88.4	62.9	<0.01	n/a	69	0.8
Linear discriminant analysis	86.0	64.7	<0.01	n/a	41	1.1
Weighted *k*-nearest neighbors	83.8	60.4	<0.01	n/a	34	2.2
*Onset/offset detection (detection rate)*						
Proposed (per-burst CNN)	97.1	82.0	—	6162	24	0.17
TCN	99.1	92.8	0.85	5010	20	0.07
LSTM-MAD	99.8	81.6	0.012	17,282	68	0.25
NMAD	100	77.5	0.003	77,634	303	0.03
TKEO	94.7	23.9	<0.001	n/a	n/a	<0.01
Double-threshold	94.5	18.6	<0.001	n/a	n/a	<0.01
AGLR	96.2	22.3	<0.001	n/a	n/a	<0.01

Abbreviations: CNN-BiLSTM: convolutional neural network with bidirectional long short-term memory; TCN: temporal convolutional network; NMAD: neural muscle-activation detector; LSTM-MAD: long short-term memory muscle-activation detector; TKEO: Teager–Kaiser energy operator; AGLR: approximated generalized likelihood ratio; Params: parameters; Mem.: memory; Inf. time: inference time; wear.: wearable; n/a: not applicable.

## Data Availability

The data presented in this study are not publicly available due to confidentiality and proprietary restrictions.

## Abbreviations

The following abbreviations are used in this manuscript:

ADC	Analogue-to-Digital Converter
CNN	Convolutional Neural Network
sEMG	surface Electromyography
ESP-NOW	Espressif peer-to-peer wireless protocol
GAP	Global Average Pooling
GLR	Generalized Likelihood Ratio
GPU	Graphics Processing Unit
IIR	Infinite Impulse Response
MAC	Multiply–Accumulate
MAE	Mean Absolute Error
MAV	Mean Absolute Value
